# Transcatheter Aortic Valve Replacement for Severe Native Aortic Valve Regurgitation: A Multicenter and International Registry

**DOI:** 10.1016/j.shj.2025.100762

**Published:** 2025-11-15

**Authors:** Sant Kumar, Ashish Pershad, David Elison, EiEi Thwe, Jacopo Farina, Ahmad Jabri, Ivan Hanson, Amr Abbas, Pedro A. Villablanca, Nezar Falluji, Simone Biscaglia, Carlo Tumscitz, Timothy Byrne, Francesco Saia, Soundos Moualla, Hursh Naik

**Affiliations:** a Department of Cardiology, Creighton University School of Medicine, Phoenix, Arizona, USA; b Department of Cardiology, St. Joseph Hospital and Medical Center, Phoenix, Arizona, USA; c Department of Cardiology, Chandler Regional Medical Center, Chandler, Arizona, USA; d Division of Cardiology, Department of Medicine, University of Washington, Seattle, Washington, USA; e Interventional Cardiology, Biltmore Cardiology, Abrazo Medical Group, Phoenix, Arizona, USA; f Cardiology Unit, Azienda Ospedaliero-Universitaria di Ferrara, Ferrara, Italy; g Division of Cardiovascular Medicine, William Beaumont University Hospital, Royal Oak, Michigan, USA; h Division of Cardiology, Center for Structural Heart Disease, Henry Ford Hospital, Detroit, Michigan, USA; i Cardiology Unit, IRCCS Azienda Ospedaliero-Universitaria di Bologna, Bologna, Italy; j Division of Cardiovascular Medicine, Dignity Health Yavapai Regional Medical Center, Yavapai, Arizona, USA

## Abstract

• Transcatheter aortic valve replacement for native aortic valve regurgitation is feasible but with modest technical success (79.8%).
• Balloon-expandable valve use is linked to lower 30-day major adverse cardiac events versus self-expanding valve in high-risk patients.
• Long-term outcomes driven by patient risk, not valve type.
• Dedicated native aortic regurgitation systems still needed to improve procedural safety.

## Introduction

Transcatheter aortic valve replacement (TAVR) is the standard of care for severe aortic stenosis but remains off-label for pure native aortic regurgitation (NAVR), where the absence of annular calcification and frequent aortic root dilation hinder anchoring and predispose to valve migration and paravalvular leak.^1^ Dedicated NAVR systems such as JenaValve and J-Valve have shown promise, yet their availability is limited, and the off-label use of commercial transcatheter heart valves (THVs) remains common.^2^ Data comparing balloon-expandable THVs (BEVs) and self-expanding THVs (SEVs) in NAVR are scarce. This multicenter, international registry evaluated procedural and clinical outcomes of contemporary BEVs and SEVs in high-risk patients with severe NAVR and identified predictors of adverse events.

## Methods

### Study Design and Population

This investigator-initiated registry included consecutive patients with severe NAVR who underwent TAVR using contemporary THVs between January 2021 and January 2025 across 6 centers in Italy and the United States. All patients were deemed high surgical risk by local heart teams. Baseline clinical, echocardiographic, and computed tomography data, procedural details, and follow-up outcomes were retrospectively collected. The study adhered to the Declaration of Helsinki, and informed consent was waived owing to its retrospective nature.

### Outcomes and Definitions

Valve type, size, and oversizing were operator determined. Native valve calcification was graded per Society of Cardiovascular Computed Tomography consensus.^3^ Outcomes were defined according to the Valve Academic Research Consortium-3 criteria.^4^ Primary endpoints were (1) technical success—a composite of successful single-valve deployment without moderate/severe regurgitation, intraprocedural death, or need for urgent surgery—and (2) major adverse cardiac events (MACE), including death, myocardial infarction, stroke, or cardiac rehospitalization. Secondary endpoints included valve embolization/migration and residual regurgitation ≥ moderate regurgitation.

### Statistical Analysis

Continuous variables were expressed as mean ± SD and compared using the Student’s or Welch t-test. Categorical variables were compared using chi-square or Fisher exact tests. Univariable logistic regression identified predictors of 30-day and long-term MACE; variables with *p* < 0.10 were entered into multivariable models. Event-free survival was assessed by Kaplan–Meier analysis with log-rank comparison. Analyses used MedCalc Software (version 12.7.7; MedCalc Software, Ostend, Belgium) with *p* < 0.05 considered significant.

## Results

### Patient Characteristics

Among 104 patients with severe NAVR treated with TAVR, 77 (74.0%) received BEVs and 27 (26.0%) received SEVs. SEV recipients were sicker with more New York Heart Association class III–IV symptoms, lower left ventricular ejection fraction, and more frequent coronary artery disease (74.1 vs. 48.1%, *p* = 0.035) (Table 1). Female sex was less common among SEV recipients (18.5 vs. 42.9%, *p* = 0.043). Computed tomography demonstrated larger sinus of Valsalva diameters (48.1 ± 6.3 mm vs. 35.2 ± 6.4 mm, *p* < 0.001) and lower left coronary heights (11.8 ± 3.7 mm vs. 14.9 ± 4.7 mm, *p* = 0.001) in SEV patients.

**Table 1:** Baseline characteristics, procedural details, and outcomes

	Total cohort (N = 104)	SEV (N = 27)	BEV (N = 77)	*p* value
Baseline characteristics				
Age, y	75.8 ± 10.6	74.5 ± 10.0	76.3 ± 10.8	0.435
Body mass index, kg/m^2^	25.8 ± 4.6	25.9 ± 3.7	25.8 ± 4.9	0.912
Female	38 (36.5)	5 (18.5)	33 (42.9)	0.043
Comorbidities				
Hypertension	88 (84.6)	23 (85.2)	65 (84.4)	0.999
Hyperlipidemia	80 (76.9)	22 (81.5)	58 (75.3)	0.698
Diabetes mellitus	32 (30.8)	11 (40.7)	21 (27.3)	0.288
Atrial fibrillation	38 (36.5)	11 (40.7)	27 (35.1)	0.768
Chronic lung disease	28 (26.9)	5 (18.5)	23 (29.9)	0.372
Home oxygen supplementation	8 (7.7)	0 (0)	8 (10.4)	0.186
Prior stroke	12 (11.5)	2 (7.4)	10 (13.0)	0.667
Prior ICD	14 (13.5)	5 (18.5)	9 (11.7)	0.571
Coronary artery disease	57 (54.8)	20 (74.1)	37 (48.1)	0.035
Prior CABG	19 (18.3)	8 (29.6)	11 (14.3)	0.137
Prior PCI	7 (6.7)	1 (3.7)	6 (7.8)	0.777
Prior pacemaker	16 (15.4)	3 (11.1)	13 (16.9)	0.685
LVEF, %	47.1 ± 12.5	43.0 ± 11.4	48.5 ± 12.6	0.041
NYHA class III or IV	89 (85.6)	27 (100)	62 (80.5)	0.010
STS risk score	5.7 ± 5.2	7.3 ± 8.7	5.2 ± 3.1	0.230
Baseline echocardiogram				
Mean aortic gradient, mmHg	12.9 ± 9.7	13.1 ± 8.1	12.8 ± 10.2	0.878
Aortic valve area, cm^2^	1.9 ± 0.9	1.9 ± 1.0	1.9 ± 0.9	1.00
Regurgitant volume, mL	69.7 ± 9.2	69.8 ± 10.1	69.6 ± 8.9	0.928
Pressure half time, ms	315.7 ± 134.9	319.5 ± 147.0	314.3 ± 131.4	0.872
Aortic regurgitation, severe	93 (89.4)	25 (92.6)	68 (88.3)	0.796
Aortic regurgitation, torrential	11 (10.6)	2 (7.4)	9 (11.7)	0.796
Baseline computed tomography				
Annular area, mm^2^	505.2 ± 138.1	519.8 ± 161.7	500.1 ± 129.6	0.571
Annular perimeter, mm	80.2 ± 10.0	77.4 ± 12.4	81.2 ± 8.9	0.152
Sinotubular junction diameter, mm	32.4 ± 5.7	31.7 ± 5.0	32.6 ± 6.0	0.449
Sinus of Valsalva diameter, mm	38.5 ± 8.5	48.1 ± 6.3	35.2 ± 6.4	<0.001
Left coronary artery height, mm	14.1 ± 4.6	11.8 ± 3.7	14.9 ± 4.7	0.001
Right coronary artery height, mm	16.5 ± 4.6	15.5 ± 3.9	16.9 ± 4.8	0.138
Aortic calcium score, AU	220.9 ± 514.5	114.7 ± 198.6	258.2 ± 583.0	0.064
Procedural details and complications				
Procedure duration, min	70.3 ± 45.7	61.2 ± 32.8	73.5 ± 49.2	0.150
Contrast volume, mL	111.9 ± 74.8	98.4 ± 99.2	116.6 ± 64.2	0.380
Embolic protection	3 (2.9)	2 (7.4)	1 (1.3)	0.164
Percent oversizing	19.0 ± 12.2	18.8 ± 6.7	19.1 ± 13.7	0.883
TEE	44 (42.3)	15 (55.6)	29 (37.7)	0.164
Procedural complications	21 (20.2)	4 (14.8)	17 (22.1)	0.560
Conversion to surgery	3 (2.9)	0 (0)	3 (3.9)	0.566
Device embolization	4 (3.8)	0 (0)	4 (5.2)	0.570
Device migration	5 (4.8)	2 (7.4)	3 (3.9)	0.603
Device thrombosis	0 (0)	0 (0)	0 (0)	-
Additional valve needed	9 (8.7)	2 (7.4)	7 (9.1)	>0.999
Technical success	83 (79.8)	23 (85.2)	60 (77.9)	0.600
Independent predictors of MACE at 30 d (adjusted analysis)				

Covariate	Odds ratio		*p* value	
SEV	REF		REF	
BEV	0.46 (0.10-0.96)		0.040	
STS risk score	1.18 (0.91-1.46)		0.318	
Diabetes mellitus	1.98 (0.33-12.33)		0.517	
Percent oversizing	0.78 (0.54-0.94)		0.005	
Left coronary height	0.66 (0.43-1.01)		0.056	
Right coronary height	0.97 (0.65-1.43)		0.870	
Aortic calcium score	0.68 (0.28-0.96)		0.023	
Independent predictors of MACE at long-term follow-up (adjusted analysis)				

Covariate	Odds ratio (95% CI)		*p* value	
SEV	REF		REF	
Age	0.95 (0.90-1.01)		0.111	
STS risk score	1.14 (1.04-1.45)		0.031	
LVEF, %	0.92 (0.81-0.98)		0.046	
Prior ICD	1.31 (0.28-6.07)		0.728	
Prior CABG	5.02 (1.54-16.40)		0.008	
Mean aortic gradient, baseline	1.14 (1.02-1.31)		0.044	
Annular perimeter diameter	0.93 (0.85-1.02)		0.136	
Sinotubular junction diameter	1.09 (0.91-1.30)		0.350	
Sinus of Valsalva diameter	1.33 (1.04-1.71)		0.024	

*Notes*. Categorical data are presented as n (%), continuous data as mean ± SD, and modeled effects as odds ratio (95% CI).

*Abbreviations*: BEV = balloon-expandable valve; CABG = coronary artery bypass graft; ICD = implantable cardioverter-defibrillator; LVEF = left ventricular ejection fraction; MACE = major adverse cardiac events; NYHA = New York Heart Association; PCI = percutaneous coronary intervention; SEV = self-expanding valve; STS = society of thoracic surgeons; TEE = transesophageal echocardiogram.

### Procedural Findings

All TAVR procedures used transfemoral access. Procedure duration (61.2 ± 32.8 vs. 73.5 ± 49.2 min, *p* = 0.150) and contrast volume (98.4 ± 99.2 vs. 116.6 ± 64.2 mL, *p* = 0.380) were similar between SEV and BEV (Table 1). Oversizing was comparable (18.8% ± 6.7% vs. 19.1% ± 13.7%, *p* = 0.883). Procedural complications occurred in 20.2% overall (14.8% SEV vs. 22.1% BEV, *p* = 0.560). Device embolization (3.8%) and migration (4.8%) rates were similar, as was the need for a second valve (8.7%). Postprocedural mild regurgitation was more frequent in SEV (22.2 vs. 6.5%, *p* = 0.051). Technical success was achieved in 79.8% (85.2% SEV vs. 77.9% BEV, *p* = 0.600).

### Short-Term Outcomes

Postimplantation hemodynamics demonstrated a significantly lower mean transvalvular gradient in SEV compared with BEV recipients (7.3 ± 3.7 mm Hg vs. 10.8 ± 6.0 mm Hg, *p* < 0.001). At 30 days, the occurrence of MACE was significantly higher in SEV (33.3 vs. 3.9%, *p* < 0.001), mainly due to cardiac rehospitalization (22.2 vs. 2.6%, *p* = 0.004). Myocardial infarction, stroke, and mortality were rare and similar between groups.

On univariable analysis, BEV use was associated with lower 30-day MACE risk (odds ratio [OR] 0.14, 95% CI 0.03–0.62; *p* = 0.009). Other predictors included diabetes mellitus (*p* = 0.025), smaller oversizing (*p* = 0.002), and lower aortic calcium score (*p* = 0.039). In multivariable analysis, BEV use (adjusted OR 0.46, 95% CI 0.10–0.96; *p* = 0.040), smaller oversizing (adjusted OR 0.78, 95% CI 0.54–0.94; *p* = 0.005), and lower calcium score (adjusted OR 0.68, 95% CI 0.28–0.96; *p* = 0.023) independently predicted 30-day MACE (Table 1).

### Long-Term Outcomes

Over a median follow-up of 412.3 days (interquartile range 202.3–791.0), 5 myocardial infarctions, 1 stroke, 12 deaths, and 24 cardiac rehospitalizations occurred. Independent predictors of long-term MACE were higher Society of Thoracic Surgeons risk score (adjusted OR 1.14, 95% CI 1.04–1.45; *p* = 0.031), lower baseline left ventricular ejection fraction (adjusted OR 0.92, 95% CI 0.81–0.98; *p* = 0.046), prior coronary bypass (adjusted OR 5.02, 95% CI 1.54–16.40; *p* = 0.008), higher baseline mean aortic gradient (adjusted OR 1.14, 95% CI 1.02–1.31; *p* = 0.044), and larger sinus of Valsalva diameter (adjusted OR 1.33, 95% CI 1.04–1.71; *p* = 0.024). Valve type was not independently associated with long-term MACE (*p* = 0.445) (Table 1). Kaplan–Meier analysis showed event-free survival was largely determined by baseline risk rather than valve type (log-rank *p* value = 0.636).

## Discussion

In this multicenter real-world cohort of patients with severe NAVR, TAVR using contemporary BEVs and SEVs was feasible but associated with suboptimal technical success (79.8%) and frequent pacemaker implantation at 30 days (20%). Notably, short-term outcomes were more favorable with BEVs, which was independently associated with a lower 30-day MACE rate compared to SEVs. However, the multivariate predictors of a favorable long-term outcome—i.e., younger age, lower Society of Thoracic Surgeons score, higher baseline ejection fraction, and absence of previous coronary artery bypass surgery—suggest long-term outcomes are primarily driven by patient comorbidities rather than by the valve type.

Device migration or embolization (8.6%) and low rates of ≥moderate regurgitation (1%) were similar to prior reports. However, the absence of annular calcification continues to challenge valve anchoring, explaining persistent risks of conduction disturbances and migration despite advances in skirt design and valve iteration. Although the overall 30-day and 1-year mortality outcomes were encouraging, the incidence of embolization or need for a second valve underscores the procedural complexity of TAVR for NAVR. These findings highlight the importance of careful anatomic screening and referral to experienced high-volume centers capable of managing device instability.

Dedicated NAVR valves such as the JenaValve Trilogy and J-Valve have achieved >90% device success and markedly lower embolization and pacemaker rates.^2^ However, regulatory limitations—including the 2025 Federal Trade Commission injunction on the Edwards Lifesciences acquisitions—may delay widespread access to these devices.^5^ Until then, the off-label use of commercial THVs remains the only option in many centers.

### Study Limitations

The study’s retrospective design introduces potential bias and limited adjudication of outcomes. The modest sample size, especially within the SEV subgroup, restricts subgroup analysis. Procedural heterogeneity and variable follow-up preclude firm conclusions about valve durability. In addition, although all patients had predominant regurgitant pathology, most exhibited some degree of aortic valve calcification; therefore, these results may not be generalizable to patients with completely noncalcified (“pure”) NAVR in whom anchoring challenges and procedural risks may be even greater.

## Conclusion

In high-risk patients with NAVR, TAVR using contemporary BEVs and SEVs is feasible and associated with acceptable technical success and low early mortality but remains limited by device migration, conduction disturbances, and rehospitalization. Long-term outcomes are primarily driven by patient and anatomic risk rather than by the valve type. These findings highlight the ongoing need for dedicated transcatheter systems and prospective trials to optimize therapy for this challenging population.

## Ethics Statement

The research reported has adhered to the relevant ethical guidelines.

## Funding

The authors have no funding to report.

## Disclosure Statement

Hursh Naik reports a relationship with 10.13039/100000046Abbott that includes consulting or advisory; reports a relationship with 10.13039/100006520Edwards Lifesciences Corporation that includes consulting or advisory. David Elison reports a relationship with Excision BioTherapeutics Inc that includes consulting or advisory; reports a relationship with Edwards that includes consulting or advisory. Pedro Villablanca reports a relationship with Edwards that includes consulting or advisory; reports a relationship with AbioMed Inc that includes consulting or advisory; reports a relationship with Shockwave Medical Inc that includes consulting or advisory; reports a relationship with Medtronic that includes consulting or advisory; reports a relationship with AngioDynamics Inc that includes consulting or advisory.

The other authors had no conflicts to declare.
